# Development of entrustable professional activities for family medicine in South Africa

**DOI:** 10.4102/phcfm.v16i1.4483

**Published:** 2024-03-29

**Authors:** Robert Mash, Louis Jenkins, Mergan Naidoo

**Affiliations:** 1 Department of Family and Emergency Medicine, Faculty of Medicine and Health Sciences, Stellenbosch University, Cape Town, South Africa; 2 Department of Primary Health Care Directorate, Family Community and Emergency Care, Faculty of Health Sciences, University of Cape Town, Cape Town, South Africa; 3 Department of Family and Emergency Medicine, George Hospital, Western Cape Department of Health, George, South Africa; 4 Department of Family Medicine, College of Health Sciences, University of KwaZulu-Natal, Durban, South Africa

**Keywords:** entrustable professional activities, family medicine, South Africa, postgraduate, training

## Abstract

South Africa is undergoing a significant shift towards implementing enhanced workplace-based assessment methodologies across various specialist training programmes, including family medicine. This paradigm involves the evaluation of Entrustable Professional Activities (EPAs) through comprehensive portfolios of evidence, which a local and national clinical competency committee then assesses. The initial phase of this transformative journey entails the meticulous development of EPAs rooted in discrete units of work. Each EPA delineates the registrar’s level of entrustment for autonomous practice, along with the specific supervision requirements. This concise report details the collaborative effort within the discipline of family medicine in South Africa, culminating in the consensus formation of 22 meticulously crafted EPAs for postgraduate family medicine training. The article intricately outlines the systematic structuring and rationale behind the EPAs, elucidating the iterative process employed in their development. Notably, this marks a groundbreaking milestone as the first comprehensive documentation of EPAs nationally for family medicine training in Africa.

## Introduction

In line with global trends in health professions education, the Colleges of Medicine of South Africa (CMSA) supported by the South African Committee of Medical Deans, have stipulated that all specialist training programmes should move towards workplace-based assessment (WBA).^1^ This includes the College of Family Physicians and the nine university-based postgraduate training programmes for family medicine.

**FIGURE 1:**
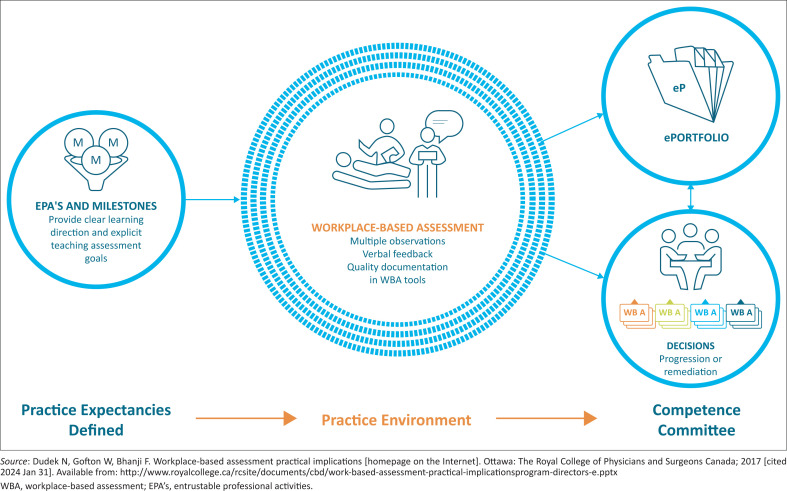
Key components of workplace-based assessment.

This article describes our journey with developing entrustable professional activities (EPAs) as a critical component of WBA (Figure 1). Other components are the development of an electronic portfolio of learning, ensuring the quality of clinical training in the workplace and establishing competency committees.^2^ Our approach to training clinical trainers has already been described in this special collection, and we hope to publish articles on the other components.^3^

## Background to workplace-based assessment

Ultimately, the national licencing examination offered by the CMSA must ensure that only competent specialists are registered to ensure the safety of patients and quality of care. Over the last 30 years, the approach to training and assessment has shifted.^1^ Initially, there was a move to outcome-based education that defined what a student should be able to do at the end of the programme. Such outcomes were often defined in terms of knowledge, skills and attitudes. Competency-based education then introduced competency frameworks (e.g. CANMeds) that defined the global competencies required of specialists.^4^ These broad competencies might be linked to a range of outcomes. However, assessment of these competencies and outcomes has often depended on once-off examinations in settings divorced from the workplace. These high-stakes summative assessments at the end of 3 or 4 years of training are limited in how much they can assess competency and, at best are pitched at the level of ‘*shows how*’ Millers Pyramid.^5^ Multiple choice questions can only assess knowledge and application of knowledge, while objective structured clinical examination (OSCE) stations are artificial situations, usually involving models or simulated patients. Many competencies are too complex or impractical to include in an OSCE station. While many of these assessments have good reliability, they often lack predictive validity for future practice.

While WBA is extremely valid at the top of Miller’s pyramid, it can be unreliable.^5^ For example, the traditional long case assessment is difficult to standardise. Programmatic assessment is an approach to WBA that tries to overcome this problem.^6^ Such WBA aims to gather multiple assessments from multiple assessors over time and in different training settings, using different assessment methods and sampling widely across the range of competencies. Each assessment or data point by itself is unreliable, but when put together as a whole they can be used to give an individual a reliable assessment. Each data point is an opportunity for feedback and ‘assessment for learning’, but when put together, it becomes an opportunity for ‘assessment of learning’.

More recently, it has been suggested that programmatic WBA should evaluate EPAs.^7^ This builds on a form of tacit assessment familiar to all senior clinicians. Clinicians are often expected to make entrustment decisions in routine clinical practice and constantly ask themselves: *Can I trust this junior doctor to see this patient, run this clinic, or perform this operation? What level of supervision do I need to give to ensure that the patient is safe and the doctor continues learning*? These are decisions about workplace activities and the level of entrustment that can be given to a specific health professional. It makes sense that WBA should assess units of work rather than more abstract educational concepts based on learning outcomes or competency frameworks.^8^

## Entrustable professional activities

In the training of family physicians in South Africa, we had previously relied on five unit standards, each broken down into numerous learning outcomes.^9^ These unit standards speak to the broad competencies required of the family physician as a leader of clinical governance, a clinician and consultant, a clinical trainer and a capacity builder. They also speak to the competencies required in community-orientated primary care and district hospitals and the underlying legal, ethical and professional attributes. In addition, we had previously defined 245 clinical skills required of family physicians.^10^ The task now was to redefine the work of a family physician in terms of EPAs.^11^

An EPA should be defined as a unit of work that makes sense in the workplace. One might, for example, imagine it as part of someone’s job description. Several EPAs need to be defined to cover all the work aspects expected from a family physician. There is no correct number of EPAs, but too few will not do justice to the complexity of the work, while too many may make it difficult for clinical trainers to cope and to assess during the training programme. Once the number and focus of the EPAs have been determined, a detailed description is written for each one. Each EPA has a standardised structure with eight components^12^:

1. Title
2. Specifications and limitations
3. Potential risks in case of failure
4. Links to the curriculum and roles of the family physician
5. Required knowledge, skills, attitudes, behaviours, experience and resources
6. Sources of evidence to support entrustment decisions
7. Entrustment level expected by end of the programme
8. Expiration date

Each of these components will be briefly outlined further in the text.

### Title

The title describes a demarcated aspect of the work expected of a family physician.

### Specification and limitations

This section specifies the professional work-related activities that are included within this EPA. It also defines the limits of this EPA and the activities that are not included. This section may also describe the various work settings within which these activities may take place. In addition, this section may link to other EPAs that contain relevant activities that are not further described here to avoid duplication.

### Potential risks in case of failure

Entrustment decisions are taken on behalf of our patients and the public. Their safety and the quality of their care need to be kept in mind when making entrustment decisions. This section reminds us of the risks if a registrar has inadequate supervision for their level of competence.

### Links to the curriculum

This section articulates how the EPA relates to the curriculum, which unit standards, learning outcomes, or a competency framework may define. In South African family medicine, we linked each EPA to the appropriate unit standard and broader roles of the family physicians.

### Required knowledge, skills, attitudes, behaviours, experience, and resources

This section describes the specific underlying knowledge, skills, attitudes, behaviours and required experience for this EPA.

#### Knowledge and skills

We combined knowledge and skills into a single list for simplicity. All skills require underlying knowledge, and we focused more on describing the application of knowledge in specific skills related to the EPA.

#### Attitudes and behaviours

Many of the expected attitudes and behaviours are generic, and we described them in a preamble to all EPAs. We expected registrars to embody the key principles of family medicine and for these principles to be visible in their behavior. For example, being person-centred, comprehensive and taking responsibility for continuity and coordination of care. We also expect registrars to demonstrate an awareness of their legal and ethical responsibilities in providing care to individuals and populations.

Professional behaviours in the workplace that are specifically related to trust, have been described as contributing to a rich entrustment decision^13^:

- Agency: Being proactive towards the needs of patients, sharing of relevant information, needs of the team, need for help, and own development.
- Reliability: Are conscientious, predictable, accountable and responsible. Do what they say and follow through on assigned tasks.
- Integrity: Are truthful, prioritise patient welfare, are person-centred and ethical.
- Capability: Are competent, have relevant knowledge and skills, efficient, adapt to changing circumstances and new tasks.
- Humility: Knows one’s limits, willing to ask for help, receptive to feedback.

#### Experience

This describes the types of rotations or allocations that are expected and which can contribute to learning in this EPA. In addition, we described the procedural or other skills from our existing list that registrars should experience. Although some skills could be linked to multiple EPAs, we tried to avoid duplication.

#### Resources

This describes some of the core and specific resources that help learning in this EPA. Certain resources were generic, applied to all EPAs, and listed in a preamble.

### Sources of evidence to support entrustment decisions

The entrustment committee will decide based on the contents of the learning portfolio. The level of entrustment for each EPA is assessed based on the available evidence. The levels of entrustment are:

1. Can observe only
2. Direct supervision (the supervisor must be next to the registrar)
3. Indirect supervision (the supervisor must be available in the facility)
4. Distant supervision (the supervisor can be available off-site at a distance)
5. Supervising others (no supervision is needed)

This assessment may be related to a progression in the programme, identifying registrars in difficulty or deciding whether this person can be certified as a family physician.

Each form or entry in the portfolio is a potential data point that can be included in the assessment. Fundamental principles regarding these data points are:

- Saturation: Are there enough entries or data points in the portfolio to make a decision about this EPA?
- Triangulation: Are there sufficient varieties of data points to make a decision about this EPA? This means from different assessors, from different contexts or settings, and from different types of assessments.
- Aggregation: Are the data points linked to a specific EPA so that the quantitative and qualitative evidence is aggregated to enable a decision?

Each EPA describes potential sources of evidence that can be linked to the EPA. For example:

1. Direct observations (e.g., mini-CEXs, DOPS [direct observation of procedural skills], others)
2. Educational meetings (with the supervisor and others)
3. Multi-source feedback
4. Periodic assessment of performance (of allocation of last 6 months)
5. Learning plans (for allocation for next 6 months)
6. Registrar reflections (on allocation of last 6 months)
7. Written assignments on workplace-based learning and practice
8. Record of allocations (relevant experience)
9. Logbook (relevant procedural and other skills)

The EPA may also define a minimum number of data points required to achieve the entrustment level by the end of the programme. For example, a minimum number of direct observations covering various activities.

### Entrustment level expected by the end of the programme

This defines the level of entrustment that needs to be obtained by the end of the programme for this EPA. Most EPAs require level 4.

### Expiration date

Once a registrar has obtained the expected level of entrustment, they do not need to be assessed again before the expiration date. Entrustment, however, does not last forever. This is because health professionals may not continue to perform the necessary skills or engage with the EPA in a way that maintains their competence. This item defines the expiration date for the expected level of entrustment. Once the registrar has obtained the expected level of entrustment, it will usually last for the duration of the training programme.

## The process of developing entrustable professional activities

A national WBA committee was established with representatives from all the university training programmes led by L.J. The committee agreed on an initial list of EPAs, developed a shared understanding of EPAs, and gained experience with completing the draft templates. We received valuable international teaching from Olle Ten Cate in a workshop organised by the South African Academy of Family Physicians. In addition, numerous workshops and consultations were held at our National Conferences. Clarity and consensus on the final list of EPAs gradually emerged. Once this was achieved the template was refined and each university was responsible for writing a selection of EPAs. The other members of the committee peer-reviewed the EPAs and finally accepted them. Altogether, 22 EPAs were created as listed in Table 1.

**TABLE 1:** The 22 entrustable professional activities for family medicine in South Africa.

Nr	EPA Title
1	Managing women and newborns in the peri-partum period
2	Managing pregnant women
3	Managing women and babies in the postnatal period
4	Managing children with undifferentiated and more specific problems
5	Managing children requiring inpatient care and procedures
6	Providing anaesthesia in the district hospital operating theatre
7	Providing anaesthesia for minor procedures
8	Managing adult and adolescent patients with chronic conditions
9	Managing adult and adolescent patients with undifferentiated problems
10	Managing patients with infectious diseases
11	Managing adults with conditions that may require surgery or procedures
12	Managing patients with mental health disorders
13	Managing patients with emergency conditions
14	Managing patients with forensic problems
15	Managing adults and children with palliative care needs
16	Managing care for older patients
17	Managing patients with impairments & rehabilitation needs
18	Supporting community-based health services
19	Supporting and providing health promotion and disease prevention services
20	Providing training and continuous professional development
21	Leading a clinical team
22	Leading clinical governance activities

EPA, entrustable professional activities; Nr, number.

## The way forward

Once the EPAs were defined, the next task was to re-design the national e-portfolio so that all the data points could be aggregated to assess the EPAs. The new e-portfolio was launched in 2024. In addition, each university and the College of Family Physicians started establishing competency committees. These committees will make summative assessments of the EPAs to guide progression in the training programmes and a final summative assessment on whether a candidate has sufficient competence to enter the national examination. How these committees will operate is still evolving. Monthly capacity-building webinars organised through the College of Family Physicians help facilitate a shared understanding of operationalising the EPAs. Plans are also in place to introduce quality assurance initiatives utilising formative assessment visits of programmes.
